# Solution structure of an arsenate reductase-related protein, YffB, from *Brucella melitensis*, the etiological agent responsible for brucellosis

**DOI:** 10.1107/S1744309111006336

**Published:** 2011-08-16

**Authors:** Garry W. Buchko, Stephen N. Hewitt, Alberto J. Napuli, Wesley C. Van Voorhis, Peter J. Myler

**Affiliations:** a Seattle Structural Genomics Center for Infectious Disease, http://www.ssgcid.org, USA; b Biological Sciences Division, Pacific Northwest National Laboratory, Richland, Washington, USA; c Department of Medicine, University of Washington, Seattle, Washington, USA; d Seattle Biomedical Research Institute, Seattle, Washington, USA; e Department of Medical Education and Biomedical Informatics and Department of Global Health, University of Washington, Seattle, Washington, USA

**Keywords:** arsenate reductases, *Brucella melitensis*, YffB, brucellosis

## Abstract

*Brucella melitensis* is the etiological agent responsible for brucellosis. Present in the *B. melitensis* genome is a 116-residue protein related to arsenate reductases (*Bm*-YffB; BR0369). Arsenate reductases (ArsC) convert arsenate ion (H_2_AsO_4_^−^), a compound that is toxic to bacteria, to arsenite ion (AsO_2_^−^), a product that may be efficiently exported out of the cell. Consequently, *Bm*-YffB is a potential drug target because if arsenate reduction is the protein’s major biological function then disabling the cell’s ability to reduce arsenate would make these cells more sensitive to the deleterious effects of arsenate. Size-exclusion chromatography and NMR spectroscopy indicate that *Bm*-YffB is a monomer in solution. The solution structure of *Bm*-YffB (PDB entry 2kok) shows that the protein consists of two domains: a four-stranded mixed β-sheet flanked by two α-helices on one side and an α-helical bundle. The α/β domain is characteristic of the fold of thioredoxin-like proteins and the overall structure is generally similar to those of known arsenate reductases despite the marginal sequence similarity. Chemical shift perturbation studies with ^15^N-labeled *Bm*-YffB show that the protein binds reduced glutathione at a site adjacent to a region similar to the H*X*_3_C*X*_3_R catalytic sequence motif that is important for arsenic detoxification activity in the classical arsenate-reductase family of proteins. The latter observation supports the hypothesis that the ArsC-YffB family of proteins may function as glutathione-dependent thiol reductases. However, comparison of the structure of *Bm*-YffB with the structures of proteins from the classical ArsC family suggest that the mechanism and possibly the function of *Bm*-YffB and other related proteins (ArsC-YffB) may differ from those of the ArsC family of proteins.

## Abstract 2

*B. melitensis* is a NIAID Category B microorganism that is responsible for brucellosis and is a potential agent for biological warfare. Here, the solution structure of the 116-residue arsenate reductase-related protein *Bm*-YffB (BR0369) from this organism is reported.

## 1. Introduction   

*Brucella melitensis* is a facultative intracellular bacterial pathogen that exhibits a host preference for goats and sheep. It is one of the *Brucella* species identified as being responsible for brucellosis, a zoonotic disease that causes abortions and stillbirths in animals and Malta fever in humans. The disease is transmitted to humans primarily *via* contact with infected animals (alive or dead) or the consumption of unpasteurized dairy products (Young, 1995 ▶). In undeveloped regions of the Mediterranean, Asia, Africa and Latin America brucellosis infections are a major problem in livestock and human populations, causing severe economic hardship (Corbel, 1997 ▶). In humans, the disease is multi-systemic and the symptoms are nonspecific, including fever, chills, malaise, dementia, fatigue, headaches, nausea, vomiting and constipation. Infections can be successfully treated with antimicrobial agents that are able to penetrate well into the host cells, often a combination of doxycycline and streptomycin or doxycycline and rifampin for prolonged periods of time (Young, 1995 ▶; Rubinstein *et al.*, 1991 ▶). There is much interest in the pathobiology of strains of *Brucella* because it is a potential agent of biological warfare and its pathogenicity is unique in that the organism does not display any obvious ‘classical’ virulence factors (Moreno & Moriyon, 2002 ▶).

Arsenic, a naturally occurring metalloid element that is frequently abundant in the environment (Messens & Silver, 2006 ▶), is a human carcinogen (Shi *et al.*, 2004 ▶) that is also toxic to most forms of life. The toxic effects arise from the reactivity of arsenic ions with protein thiols. To counter the toxic effects, a family of arsenic detoxification enzymes has evolved that convert arsenate ion (H_2_AsO_4_^−^), the highly reactive form of arsenic, to arsenite ion (AsO_2_^−^), a compound that may be effectively transported outside the cell (Mukhopadhyay *et al.*, 2002 ▶). In *B. melitensis*, YffB, a protein with marginal sequence similarity to the classical family of arsenate reductases (ArsC), is found that may play a role in reducing arsenate (DelVecchio *et al.*, 2002 ▶). This 13.5 kDa protein (*Bm*-YffB) is a potential drug target because if arsenate reduction is this protein’s major biological function, con­tributing to the organism’s virulence, then disabling this protein and the cell’s ability to reduce arsenate would make *B. melitensis* more sensitive to the deleterious effects of endogenous arsenate. Towards understanding the biological function of *Bm*-YffB and providing a blueprint for structure-based drug design (Myler *et al.*, 2009 ▶) based on this protein, the solution structure of *Bm*-YffB was determined. Its thermostability was measured by CD spectroscopy and its structure was compared with those of a similar protein, *Pseudomonas aeruginosa* YffB (*Pa*-YffB; PDB entry 1rw1; Teplyakov *et al.*, 2004 ▶), and a protein known to reduce arsenate, *Escherichia coli* ArsC (*Ec*-­ArsC; PDB entry 1id9; Martin *et al.*, 2001 ▶).

## 2. Materials and methods   

### 2.1. Cloning, expression and purification   

The Bm-*Yffb* gene (BR0369; YP_4138591.1) was amplified using the genomic DNA of *B. melitensis* biovar Abortus 2308 and the oligonucleotide primers 5′-GGGTCCTGGTTCGATGAGTGTGA­CCATTTACGGCATC-3′ (forward) and 5′-CTTGTTCGTGCTG­TTTATTATAGCTTAAAATAAGCTTCATACTGCG-3′ (reverse) (Invitrogen, Carlsbad, California, USA). The amplified Bm-*YffB* gene was then gel-purified, treated with T4 DNA polymerase and annealed into the *Nru*I/*Pme*I-digested expression vector AVA0421 at a site that provided a 21-residue tag containing six consecutive histidine residues (MAHHHHHHMGTLEAQTQGPGS-) at the N-­terminus of the expressed protein (Choi *et al.*, 2011 ▶). The recombinant plasmid was transformed into *E. coli* BL21(DE3)R3-pRARE2 cells (a gift from SGC Toronto, Toronto, Ontario, Canada) using a heat-shock method. Uniformly ^15^N- and ^15^N-,^13^C-labeled *Bm*-­YffB was obtained by growing the transformed cells (310 K) in minimal medium (Miller) containing ^15^NH_4_Cl (1 mg ml^−1^) and d-­[^13^C_6_]-glucose (2.0 mg ml^−1^) supplemented with FeCl_3_ (50 µg ml^−1^) and the antibiotics chloramphenicol (35 µg ml^−1^) and ampicillin (100 µg ml^−1^). Once the cells reached an OD_600_ of ∼0.8, the cells were cooled to 298 K and protein expression was induced with iso­propyl β-d-1-thiogalactopyranoside (0.026 µg ml^−1^). After approximately 5 h, the cells were harvested by mild centrifugation and frozen at 193 K. The frozen pellet was later thawed and resuspended in ∼35 ml lysis buffer (0.3 *M* NaCl, 50 m*M* sodium phosphate, 10 m*M* imidazole, pH 8.0) brought to 0.2 m*M* phenylmethylsulfonyl fluoride (PMSF) prior to three passes through a French press (SLM Instruments, Rochester, New York, USA). Following 60 s sonication (SLM Instruments, Rochester, New York, USA) the cell debris was pelleted by centrifugation at 25 000*g* for 1 h in a JA-20 rotor (Beckman Instruments, Fullerton, California, USA). The supernatant was then passed through a 0.45 µm syringe filter and applied onto an Ni–NTA affinity column (Qiagen, Valencia, California, USA) containing ∼25 ml resin. The column was washed stepwise by gravity with 40 ml buffer (0.3 *M* NaCl, 50 ml sodium phosphate pH 8.0) containing increasing con­centrations of imidazole (5, 10, 20, 50 and 250 m*M*). *Bm*-YffB eluted primarily in the 250 m*M* imidazole wash. Following exchange into 3C cleavage buffer by overnight dialysis in 4 l cleavage buffer (150 m*M* NaCl, 20 m*M* Tris–HCl, pH 8.4) the protein was concentrated to ∼2 ml (Amicon Centriprep-10) and the N-terminal polyhistidine tag was removed by overnight incubation with 3C protease (1 µg per 50 µg target protein) at 279 K (Bryan *et al.*, 2011 ▶). Using a flow rate of 1.0 ml min^−1^, the reaction solution was then loaded onto a Superdex 75 HiLoad 16/60 column (GE Healthcare, Piscataway, New Jersey, USA) to simultaneously purify the protein and exchange it into NMR buffer (100 m*M* NaCl, 20 m*M* Tris–HCl, 1.0 m*M* dithiothreitol, pH 7.1). The band containing *Bm*-YffB (retention time 78 min) was collected and the volume was reduced (Amicon Centriprep-10) to generate NMR samples in the 1–2 m*M* range (Lowry analysis). SDS–PAGE analysis of the final NMR samples showed the protein to be greater than ∼95% pure.

### 2.2. Circular dichroism spectroscopy   

An Aviv Model 410 spectropolarimeter (Lakewood, New Jersey, USA) calibrated with an aqueous solution of ammonium d-(+)-camphorsulfonate was used to collect circular dichroism data from a 0.05 m*M* *Bm*-YffB sample in NMR buffer in a quartz cell of 0.1 cm path length. A thermal denaturation curve was obtained by recording the ellipticity at 216 nm in 2.0 K intervals from 283 to 353 K. A quantitative estimation of the melting temperature, *T*_m_, was obtained by taking a first derivative of the thermal denaturation curve using the Aviv software (Greenfield, 2006 ▶). Steady-state wavelength spectra for *Bm*-YffB were recorded in 0.5 nm increments between 200 and 260 nm at 298 and 353 K. Each reported steady-state wavelength spectrum was the result of averaging two consecutive scans with a bandwidth of 1.0 nm and a time constant of 1.0 s. These spectra were processed by subtracting a blank spectrum from the protein spectrum and then automatically line-smoothing the data using the *Aviv* software.

### 2.3. Nuclear magnetic resonance spectroscopy   

Varian 800-, 750- and 600-Inova spectrometers equipped with ^1^H/^13^C/^15^N triple-resonance probes and pulse-field gradients were used to collect the NMR data required for resonance assignments and structure determination. The NMR data, which were collected from 1–2 m*M* samples at 293 K, were processed with *Felix*2007 (Felix NMR Inc., San Diego, California, USA) and analyzed with *Sparky* (v.3.115; Goddard & Kneller, 2008 ▶). Assignments of the ^1^H, ^13^C and ^15^N chemical shifts for the backbone and side-chain resonances were made from standard 2D ^1^H–^15^N HSQC, ^1^H–^13^C HSQC, HBCBC­GCDHD and HBCBCGCDCHE experiments and 3D HNCACB, CBCA(CO)NH, HNCO, HCC-TOCSY-NNH and CC-TOCSY-NNH experiments using Varian *Protein Pack* pulse programs. Chemical shifts were referenced to DSS (DSS = 0 p.p.m.) using indirect methods (Wishart *et al.*, 1995 ▶). Distance restraints were obtained from a suite of 3D ^13^C- and ^15^N-edited NOESY-HSQC experiments using a mixing time of 80 ms. Deuterium-exchange studies were performed by lyophilizing a ^15^N-­labeled NMR sample, re-dissolving it in 99.8% D_2_O and immediately collecting ^1^H–^15^N HSQC spectra 10, 20 and 60 min after exchange. An overall rotational correlation time, *t*_c_, was estimated from backbone-amide ^15^N *T*_1_/*T*_1ρ_ ratios (Farrow *et al.*, 1994 ▶; Buchko *et al.*, 2008 ▶). A chemical shift perturbation experiment was performed by adding aliquots of reduced glutathione in NMR buffer (50 m*M*) to a 0.5 m*M* sample of ^15^N-­labeled *Bm*-YffB. Following gentle agitation, ^1^H–^15^N HSQC spectra were collected at glutathione:*Bm*-YffB molar ratios of 0.3:1, 0.6:1, 1:1 and 2:1.

### 2.4. Structure calculations   

The chemical shifts for *Bm*-YffB were assigned using conventional methods (Cavanagh *et al.*, 1996 ▶) and were deposited in the Biological Magnetic Resonance Data Bank (BMRB) under accession No. 16517. Using these ^1^H, ^13^C and ^15^N chemical shift assignments and the peak-picked data from ^13^C- and ^15^N-edited NOESY-HSQC experiments as initial inputs, structure calculations were performed iteratively using *CYANA* (v.2.1; Güntert, 2004 ▶). 184 dihedral angle restraints for both ϕ and ψ were introduced on the basis of the elements of secondary structure identified in the early structural ensembles and *TALOS* calculations (Cornilescu *et al.*, 1999 ▶). Near the end of the iterative process, 84 hydrogen-bond restraints (1.8–2.0 and 2.7–3.0 Å for the NH—O and N—O distances, respectively) were introduced into the structure calculations on the basis of proximity in early structure calculations and the observation of slowly exchanging amides in the deuterium-exchange experiment. On the basis of the chemical shift difference between the proline ^13^C^β^ and ^13^C^γ^ atoms (Schubert *et al.*, 2002 ▶), Pro93 was placed in the *cis* conformation. From the final set of 100 calculated structures, the 20 with the lowest target function were selected and this ensemble was deposited in the Protein Data Bank (PDB) under PDB code 2kok. Structural quality was assessed using the *Protein Structure Validation Suite* (*PSVS*; v.1.3; Bhattacharya *et al.*, 2007 ▶). Note that the deposited structures were not refined with explicit water (Linge *et al.*, 2003 ▶) because these calculations con­tinuously introduced unfavorable steric clashes into the structures despite numerous attempts to adjust the parameters. A summary of the structure statistics is provided in Table 1 ▶.

The amino-acid sequence of *Bm*-YffB deposited in the PDB and BMRB is numbered sequentially, Gly1–Leu120, starting with the four non-native residues (GPGS-) that remained after 3C protease treatment. However, here the first four non-native residues are numbered sequentially with asterisks (Gly1*–Ser4*) and the first native residue, Met5 in the PDB and BMRB depositions, is labeled Met1.

## 3. Results and discussion   

### 3.1. Solution structure of *Bm*-ArsC   

**Figure 1:**
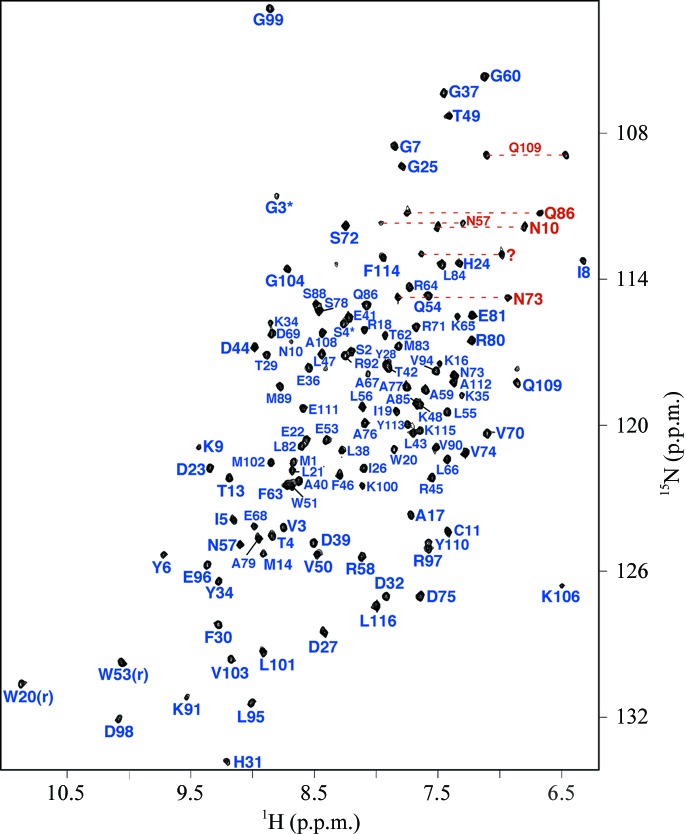
Assigned ^1^H–^15^N HSQC spectrum of double-labeled *Bm*-YffB collected at 293 K in NMR buffer (100 m*M* NaCl, 20 m*M* Tris–HCl, 1.0 m*M* DTT, pH 7.1) at a ^1^H resonance frequency of 750 MHz. Side-chain –NH_2_ resonances are indicated by dashed horizontal lines (red) and the exchangeable ring resonances for Trp20 and Trp53 are identified with an ‘r’.

**Figure 2:**
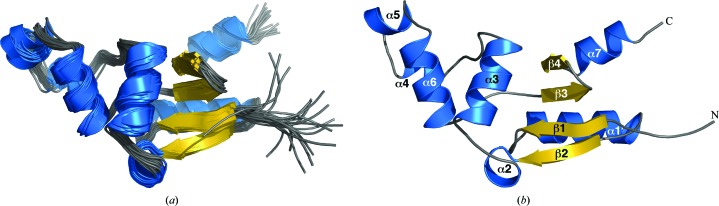
(*a*) Superposition of the cartoon representations of the ensemble of structures calculated for *Bm*-YffB (PDB entry 2kok), with α-helices colored blue and β-strands colored gold. (*b*) Cartoon representation of the structure most similar to the average structure of the ensemble, with the four β-strands and seven α-helices labeled.

The elution time of *Bm*-YffB on a Superdex 75 HiLoad 16/60 column was within a range consistent with a monomeric ∼14 kDa protein (data not shown). Such a conclusion was corroborated by the experimentally estimated rotational correlation time determined for *Bm*-YffB, 9.1 ± 0.2 ns (293 K), a value that is more consistent with a monomeric ∼14 kDa protein than an ∼28 kDa dimer (Bhattacharjya *et al.*, 2004 ▶). The line widths and chemical shift dispersion of the ^1^H–^15^N HSQC spectrum for *Bm*-YffB, shown in Fig. 1 ▶, were also characteristic of a folded monomeric protein with a molecular weight in the 15 kDa range. As illustrated in Fig. 1 ▶, 121 of the expected 123 amide resonances for *Bm*-YffB [120 − (6 prolines + Gly1*)] were assigned in the ^1^H–^15^N HSQC spectrum. Amide cross-peaks for Asp16 and Phe109 were not unambiguously identified. On the basis of these amide assignments and extensive assignment of the ^13^C^α^ and side-chain proton and carbon chemical shifts (BMRB ID 16517), an ensemble of structures was calculated (Fig. 2 ▶ *a*) that satisfied all of the available experimental NMR data (NOEs, chemical shifts, deuterium-exchange experiments and *TALOS* calculations).

As summarized in Table 1 ▶, a total of 1490 interproton distance restraints, 84 hydrogen-bond restraints and 188 dihedral angle restraints were used in the final structure calculations. Each member of the final ensemble of 20 calculated structures agreed well with the experimental data, with no upper limit violation of greater than 0.05 Å and no torsion-angle violation of greater than 2°. Analysis of the ensemble with the *PSVS* validation-software package (Bhattacharya *et al.*, 2007 ▶) also showed that the quality of the structures in the final ensemble was good. The Ramachandran statistics for all residues in the ensemble were overwhelmingly in acceptable space [90% of the (ϕ, ψ) pairs for *Bm*-YffB were found in the most favored regions and 10% were within additionally allowed regions] and all the structure-quality *Z* scores were acceptable (>−5).

**Figure 3:**
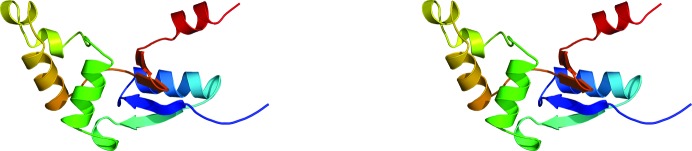
Stereoview showing a cartoon representation of the structure most similar to the average structure in the ensemble, with the protein rainbow-colored (ROYGBIV) from the N-terminus to the C-terminus.

**Figure 4:**
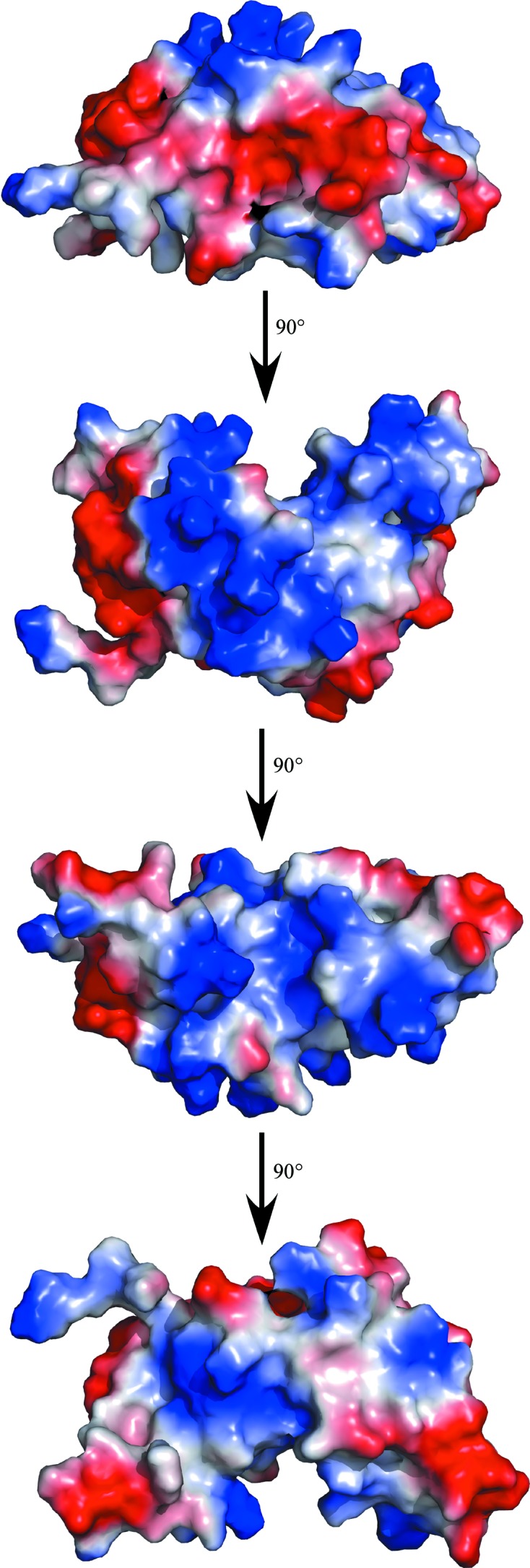
Maps generated using *PyMOL* (DeLano, 2002 ▶) of the electrostatic potential at the solvent-accessible surface of *Bm*-YffB. The long axis of the protein is illustrated and is sequentially rotated 90° about the horizontal axis four times.

The final set of 20 calculated structures in the ensemble converge well, as shown mathematically by the statistics in Table 1 ▶ and visually by the superposition in Fig. 2 ▶(*a*). The r.m.s.d.s of the structured core regions (Val3–Ile8, Cys11–Ala59, Thr61–Asp98 and Lys100–Lys115) in the ensemble from the mean structure are 0.7 Å for the backbone atoms (N—C^α^—C=O) and 1.2 Å for all heavy atoms. Fig. 2 ▶(*b*) illustrates the single structure in the ensemble that is nearest to the mean structure and Fig. 3 ▶ shows a stereoview of this single structure. The protein consists of two domains: a four-stranded mixed β-sheet flanked by two α-helices on one face and an α-helical bundle. The α/β domain is composed of a β4–β3–β1–β2 β-sheet, with β4–β3–β1 aligned antiparallel and β1–β2 aligned parallel. α-Helix 1 (Asp12–His24) is tucked in behind β1–β2, and α7 (Pro107–Phe114) is tucked in behind β4–β3. The α/β domain is characteristic of the fold of thioredoxin-like proteins and usually contains a *cis*-proline on the N-­terminal side of β3 that plays a role in the integrity of the active site (Martin, 1995 ▶). *Bm*-YffB also contains a proline, Pro93, at this position and analysis of the ^13^C^β^ and ^13^C^γ^ chemical shifts for this residue indicates that it is also in the *cis* conformation (Table 2 ▶; Schubert *et al.*, 2002 ▶). The other domain in *Bm*-YffB is an α-helical bundle dominated by α3 (Ala40–Thr49) and α6 (Ala76–Ala85), with three short helices [α2 (Tyr33–Glu36), α4 (Thr61–Lys65) and α5 (Glu68–Ser72)] around them. Hydrophobic interactions between the side chains of residues on the interface of these two domains, including Phe46 (α3), Thr49 (α3), Tyr6 (β1) and Leu101 (β4), assist in holding the domains together. As shown in Fig. 4 ▶, the protein con­tains a polarized distribution of charges on its surface, with a dominance of positive surfaces. Such a distribution of charges has been observed in the ArsC protein family, as well as for *Pa*-YffB, and would favor the binding of anions such as arsenate (Teplyakov *et al.*, 2004 ▶).

### 3.2. Circular dichroism profile and thermal stability of *Bm*-ArsC   

**Figure 5:**
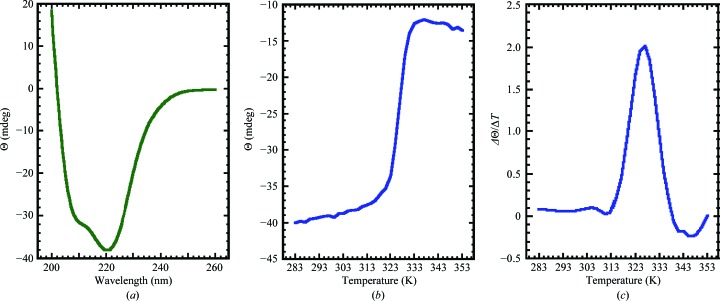
(*a*) Circular dichroism steady-state wavelength spectrum for *Bm*-YffB (0.05 m*M*) in NMR buffer collected at 298 K. (*b*) The CD thermal melt for *Bm*-YffB obtained by measuring the ellipticity at 216 nm in 2.0 K intervals between 283 and 353 K. (*c*) The first derivative of the thermal melt curve shows that the protein has a melting temperature of 326.6 K.

Circular dichroism (CD) spectroscopy is very sensitive to changes in a protein’s backbone and, consequently, is a powerful tool to rapidly probe the conformation of proteins in solution and to assess the effect of variables such as pH, salt content and temperature on the structure of a protein (Woody, 1974 ▶). Fig. 5 ▶(*a*) shows the steady-state CD spectrum of *Bm*-YffB collected at 298 K. The dominant feature of the spectrum is characteristic of α-helical secondary structure: a double minimum at approximately 220 and 208 nm and an extrapolated maximum around 195 nm (Holzwarth & Doty, 1965 ▶; Greenfield, 2006 ▶). Such an observation is expected given the amount of helical structure (46%) observed in the solution structure of the protein (Figs. 2 ▶ and 3 ▶). Note that the double minimum is skewed and is more intense around 220 nm, which is likely to be due to the contributions of other elements of secondary structure to the CD steady-state spectrum.

By monitoring the increase in the ellipticity at a specific wavelength with increasing temperature, the thermal stability of a protein may be measured and a melting temperature (*T*_m_) estimated for the transition between a structured and an unstructured state (Karantzeni *et al.*, 2003 ▶). As shown in Fig. 5 ▶(*b*), a gradual increase in ellipticity at 220 nm is observed for *Bm*-YffB up to ∼323 K, followed by a more rapid increase in ellipticity that tails off and plateaus at ∼333 K. Visual inspection of the sample after heating to 353 K showed evidence of precipitation, indicating that the unfolding was irreversible and that the CD data may not be analyzed thermodynamically (Karantzeni *et al.*, 2003 ▶). However, a quantitative estimation of the *T*_m_ for this transition may still be obtained by assuming a two-state model and taking a first derivative of the curve shown in Fig. 5 ▶(*b*) (Greenfield, 2006 ▶). The maximum of this first derivative, shown in Fig. 5 ▶(*c*), is 326.6 K.

### 3.3. Comparison with related structures   

**Figure 6:**
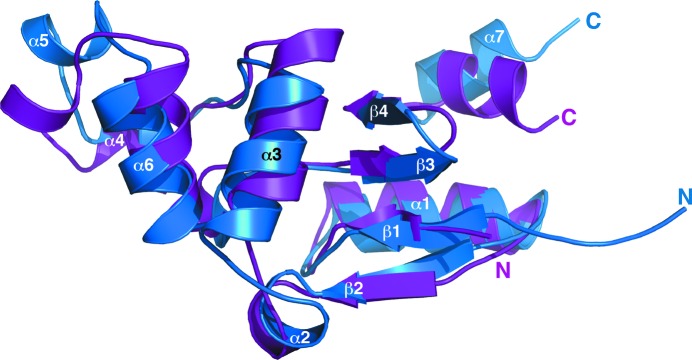
Superposition of the structure closest to the mean for *Bm*-YffB (PDB entry 2kok, blue) on the crystal structure determined for *Pa*-YffB (PDB entry 1rw1, magenta; Teplyakov *et al.*, 2004 ▶) using the program *SuperPose* (Maiti *et al.*, 2004 ▶).

The PDB was searched for structures similar to *Bm*-YffB using the *DALI* search engine (Holm & Rosenström, 2010 ▶). The search indicated that the structure of *Bm*-YffB was most similar (*Z* score = 13.4) to that of the conserved hypothetical protein YffB from *P. aeruginosa* (PDB entry 1rw1; Teplyakov *et al.*, 2004 ▶). This similarity is evident in Fig. 6 ▶, which shows a superposition of the two structures with the program *SuperPose* (Maiti *et al.*, 2004 ▶). It is evident that both proteins have the same number of β-strands and α-helices organized into a similar two-domain structure. Indeed, using *Bm*-YffB residues Ser2–Lys115 the backbone r.m.s.d. is 3.78 Å between *Bm*-YffB and the equivalent region in *Pa*-YffB. Such similarity between the structures of the two proteins is reasonable given that the amino-acid sequences of the two proteins are 56% identical and 70% similar.

After *Pa*-YffB, the *DALI* search identified a number of proteins with *Z* scores between 11.3 and 10.0 that were annotated as arsenate reductases (ArsC) or Spx regulatory proteins. While the structure of *Bm*-YffB produced high *DALI* *Z* scores with these two types of proteins, the sequence identity between *Bm*-YffB and these proteins was only 14–27%. The Spx protein is a global transcription regulatory protein that does not bind DNA as part of its regulatory role but instead binds to the α-subunit of RNA polymerase to control gene expression in response to disulfide-stress conditions (Nakano *et al.*, 2005 ▶). In Spx it is hypothesized that disulfide-bond formation in a C*XX*C motif triggers events that result in an increase in the cellular levels of thioredoxin and thioredoxin reductase (Nakano *et al.*, 2003 ▶). Because *Bm*-YffB is a monomer and contains only a single cysteine residue, it is unlikely that it functions as a regulatory protein using a mechanism similar to that which may be employed by Spx. On the other hand, *Bm*-YffB contains a sequence that is somewhat similar to the H*X*_3_C*X*_3_R catalytic sequence motif that is important for arsenic detoxification activity in the arsenate-reductase family of proteins (ArsC; Martin *et al.*, 2001 ▶); hence, it is more likely that *Bm*-YffB has a function related to arsenate reduction.

**Figure 7:**
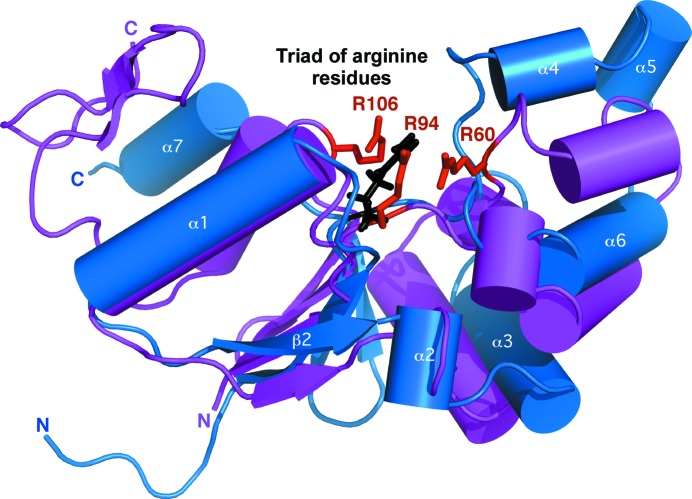
Superposition of the structure closest to the mean for *Bm*-YffB (PDB entry 2kok, blue) on the crystal structure determined for *E. coli* ArsC (PDB entry 1id9, magenta; Martin *et al.*, 2001 ▶) using the program *SuperPose* (Maiti *et al.*, 2004 ▶). The side chains of the three arginine residues in *Ec*-ArsC (Arg60, Arg94 and Arg107) that form a thiasahydroxyl adduct that is essential for enzymatic function are labeled and colored red. The only residue in *Bm*-YffB that is equivalent to those in the arginine triad of *Ec*-ArsC is Arg96; this side chain is colored black.

Fig. 7 ▶ shows a superposition of a representative structure of *Bm*-YffB (PDB entry 2kok) with the crystal structure of *E. coli* ArsC (*Ec*-ArsC) in the native form (PDB entry 1id9; Martin *et al.*, 2001 ▶) using the program *SuperPose* (Maiti *et al.*, 2004 ▶). Apart from an extra two-stranded β-sheet at the C-terminus of *Ec*-ArsC, the two structures are generally similar to each other: a four-stranded mixed β-sheet is flanked by a number of α-helices that generate a similar overall fold. One major difference between the two structures, highlighted in Fig. 7 ▶, is the presence of a triad of arginine residues, Arg60, Arg94 and Arg106 (red), in *Ec*-ArsC that is absent in *Bm*-YffB. These three arginines have been reported to be essential for enzymatic function, forming a thiasahydroxyl adduct when bound to arsenate (Martin *et al.*, 2001 ▶). In the *Bm*-YffB structure only one arginine, Arg96 (black), is found in this area. Such a major structural difference suggests a difference in substrate specificity between *Bm*-YffB and *Ec*-ArsC and it has been postulated that the ArsC-YffB family of proteins might be able to bind glutathione in the absence of arsenate (Teplyakov *et al.*, 2004 ▶).

### 3.4. Chemical shift perturbation studies with reduced glutathione   

**Figure 8:**
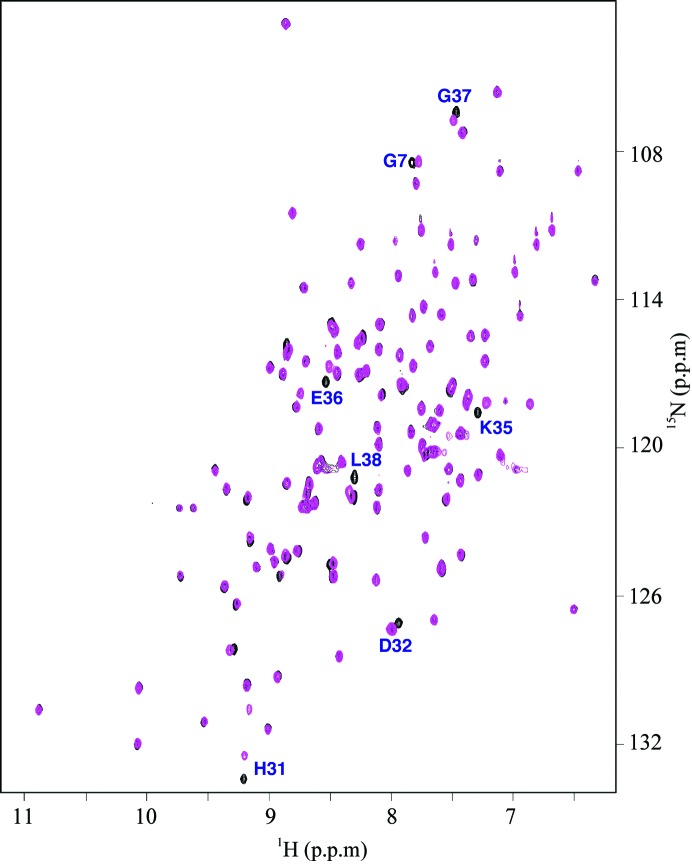
Overlay of the ^1^H–^15^N HSQC spectrum of ^15^N-labeled *Bm*-YffB (black) on the spectrum collected in the presence of a 2:1 molar ratio of glutathione:*Bm*-YffB (magenta). Residues that were significantly perturbed are labeled. The spectrum at an ∼1:1 molar ratio of glutathione:*Bm*-YffB was similar to that shown here at a 2:1 molar ratio. Data were collected at a proton resonance frequency of 600 MHz at 293 K.

**Figure 9:**
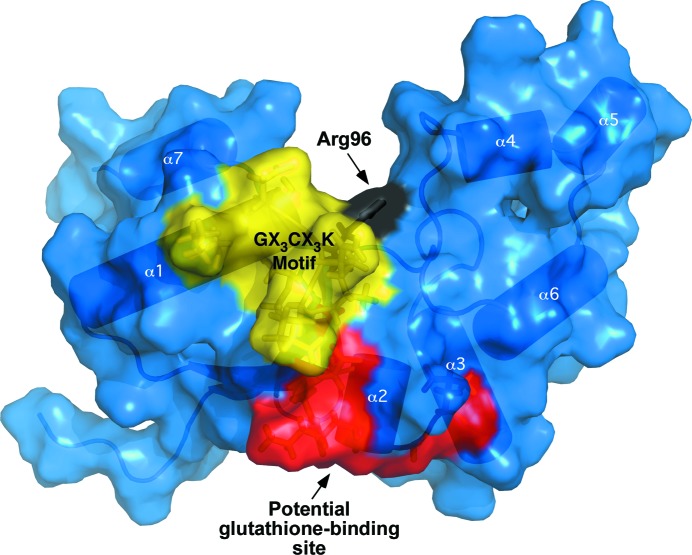
Surface representation of the structure of *Bm*-YffB shown with a labeled transparent cartoon representation. Regions that may potentially be important for the function of the protein are labeled. The lone equivalent residue to those present in the arginine triad of *Ec*-ArsC, Arg96, is colored black, residues in the potential G*X*_3_C*X*_3_K catalytic sequence motif are colored yellow and the residues perturbed by the addition of reduced glutathione (labeled in Fig. 8 ▶) are colored red.

In support of the hypothesis that the ArsC-YffB family of proteins may bind glutathione in the absence of arsenate ions, it has been shown by mass spectrometry that *P. aeruginosa* YffB can bind glutathione (Teplyakov *et al.*, 2004 ▶). One of the advantages of structure determination by NMR-based methods is that following the chemical shift assignment of the ^1^H–^15^N HSQC spectrum, not only is it possible to quickly detect ligand binding to the protein by chemical shift perturbation experiments, but, it is also possible to map the location of the binding interaction on the three-dimensional structure of the protein (Buchko *et al.*, 1999 ▶; Zuiderweg, 2002 ▶). This is possible because protein–ligand interactions often manifest as changes in the chemical environment of the nuclei at the interface of ligand binding which are accompanied by perturbations in the measurable chemical shifts of the backbone ^1^H^N^ and ^15^N resonances. Upon identifying the resonances that experience a binding-dependent chemical shift or intensity perturbation, it is possible to map the location of the binding site onto the structure of the protein. Fig. 8 ▶ is the result of such an experiment following the titration of reduced glutathione into a ^15^N-­labeled sample of *Bm*-YffB. At a 2:1 molar ratio of reduced glutathione:*Bm*-YffB, a subset of seven resonances are observed to shift. In Fig. 9 ▶ these perturbed resonances are mapped onto the structure of *Bm*-YffB and it is observed that they all cluster into one area (red) adjacent to the potential G*X*_3_C*X*_3_K catalytic sequence motif (yellow). The latter observation adds support to the hypothesis that *Bm*-YffB and other proteins in the YffB family may function as glutathione-dependent thiol reductases.

## 4. Conclusions   

The solution structure determined for *Bm*-YffB is similar to the crystal structure reported for the YffB protein from *P. aeruginosa*: a four-stranded mixed β-sheet flanked by two α-helices on one side and an α-helical bundle. While the protein sequences of *Bm*-YffB and *Pa*-­YffB are marginally similar to the amino-acid sequences of classical arsenate reductases (ArsC), comparison of the structures of YffB proteins and ArsC proteins suggest that the substrate specificities and mechanisms of these two families of proteins may differ. Chemical shift perturbation studies with reduced glutathione corroborate the hypothesis that YffB proteins may function as glutathione-dependent thiol reductases. Further biochemical and biophysical studies will be necessary in order to identify the substrate and work out the details of the mechanism before *Bm*-YffB may be exploited for structure-based drug design. The solution structure and bio­physical data presented here for *Bm*-YffB will facilitate these efforts.

## Supplementary Material

PDB reference: *Bm*-YffB, 2kok

## Figures and Tables

**Table 1:** Summary of the structural statistics for *Bm*-YffB All statistics are for the 20-structure ensemble deposited in the PDB (2kok).

Restraints for structure calculations	
Total NOEs	1490
Intraresidue NOEs	436
Sequential (*i*,*i* + 1) NOEs	449
Medium-range (*i*,*i* + *j*; 1 *j* 4) NOEs	282
Long-range (*i*,*i* + *j*;*j*> 4) NOEs	323
-angle restraints	94
-angle restraints	94
Hydrogen-bond restraints	84
Structure calculations	
No. of structures calculated	100
No. of structures used in ensemble	20
Structures with restraint violations	
Distance restraint violations> 0.05	0
Dihedral restraint violations> 2	0
R.m.s.d. from mean† ()	
Backbone NCCO atoms	0.69 0.16
Heavy atoms	1.19 0.11
Ramachandran plot summary for selected residues (from *PROCHECK*)‡	
Most favored regions (%)	89.8
Additionally allowed regions (%)	10.2
Generously favored regions (%)	0.0
Disallowed regions (%)	0.0
Global quality scores for ordered residues‡ §	
*PROCHECK* (all)	2.84 (0.48)
*PROCHECK* (, )	0.28 (0.15)
*MolProbity* clash score	0.86 (13.91)

† Calculated for the ordered residues Val3Ile8, Cys11Ala59, Thr61Asp98 and Lys100Lys115.

‡ Calculated for the central ordered core, Ser2Ile8 and Cys11Lys115, using *PSVS*.

§ *Z* scores; values in parentheses are raw values.

**Table 2:** Chemical shift difference between the proline ^13^C and ^13^C atoms in *Bm*-YffB

Residue	(p.p.m.)
Pro2*	5.0
Pro51	5.0
Pro67	4.6
Pro87	4.2
Pro93	8.7
Pro107	4.2
Average for *trans*-Pro	4.51 1.17†
Average for *cis*-Pro	9.64 1.27†

† Average values obtained from Schubert *et al.* (2002 ▶).
